# Luseogliflozin Additively Enhances the Glucose-Lowering Effect of an Incretin Modulator in a High-Carbohydrate Diet

**DOI:** 10.7759/cureus.30410

**Published:** 2022-10-17

**Authors:** Gendai Lee

**Affiliations:** 1 Diabetes and Endocrinology, Diabetes and Kidney Medical Corporation Gendai Clinic, Kitakyushu, JPN

**Keywords:** intermittently scanned continuous glucose monitoring, type 2 diabetes mellitus, high carbohydrate, incretin modulator, sglt2 inhibitor

## Abstract

Background and objectives

Sodium/glucose co-transporter 2 inhibitors (SGLT2i) have been shown to have a glucose-lowering effect related to carbohydrate intake. It has also been reported that the combined effect of incretin modulators and SGLT2i is useful in improving blood glucose and reducing blood glucose variability. However, there have been no reports examining the effects of these two drugs together and while considering carbohydrate intake in an outpatient setting. In the present study, Hi-Speed Food Analysis was used to assess the exact intake of carbohydrates, and the glucose-lowering effects of luseogliflozin, an SGLT2i, and incretin modulators were examined under high- and low-carbohydrate intakes.

Methods

Thirty-five enrolled diabetic patients continued their regular medications for one week. All patients took luseogliflozin in the second week for seven days. During the two weeks, ingested carbohydrates were accurately calculated by Hi-Speed Food Analysis. The glucose-lowering effect of luseogliflozin with and without incretin modulators was checked according to the amount of ingested carbohydrates. A general linear model (GLM) was used to analyze the effect of luseogliflozin with or without an incretin modulator, with carbohydrate intake as a confounding factor.

Results

Luseogliflozin had an additive effect in patients who had taken the incretin modulator. There was a significant decrease in the time above range (TAR) with glucose above 140 mg/dL as expressed as TAR(min140), and this effect was affected by carbohydrate intake.

Conclusions

The glucose-lowering effect of luseogliflozin was enhanced with high-carbohydrate intake more than with low-carbohydrate intake. In this study, the observed number was small; however, combined treatment with an incretin modulator and luseogliflozin had an additive effect in high- versus low-carbohydrate intake, indicating the possible effectiveness of the combined therapy.

## Introduction

Emerging sodium/glucose co-transporter 2 inhibitors (SGLT2i) have been introduced as therapies for type 2 diabetes mellitus (T2DM) and have been found to be clinically effective [1]. SGLT2i have various effects in treating T2DM, including the reduction of hyperglycemia, attenuation of glucose toxicity, and decreased body weight by eliminating urinary glucose. In addition, they show a reduced rate of hospitalization for heart failure, cardiovascular disease mortality, all-cause mortality, and progression of diabetic kidney disease [2]. On the other hand, SGLT2i lead to endogenous glucose production, which is most likely driven by increased glucagon production.

Luseogliflozin is a highly selective SGLT2i that was approved in Japan for treating T2DM [3,4].

Dipeptidyl peptidase 4 inhibitors (DPP4i) enhance postprandial insulin secretion and suppress glucagon secretion by preventing the degradation of endogenously released incretins such as glucagon-like peptide 1 (GLP-1) and glucose-dependent insulinotropic polypeptide (GIP), which are two intestinal peptides whose concentrations increase after food intake, thus playing essential roles in glucose homeostasis [5]. Glucagon-like peptide-L receptor agonist (GLP-IRA) is a glucose-dependent, insulin-mediated plasma glucose-dependent insulin secretion and suppression of glucagon, suppresses diurnal variation in blood glucose levels by delaying gastric emptying, and has a hypoglycemic effect by delaying gastric emptying [6]. Efficacy and safety have also been demonstrated in combination therapy with liraglutide or sitagliptin and sulfonylureas in Japanese patients with T2DM in primary care [7]. The combination with an SGLT2i and incretin modulator shows additional benefits for glycemic control [8]. In a meta-analysis comparing SGLT2i and incretin modulator combination therapy to SGLT2i alone or as an add-on to metformin, the combination therapy with an incretin modulator was found to provide additional benefit in the treatment of T2DM, especially in Asians [9]. Combined therapy with an SGLT2i and a DPP4i reduced glycemic fluctuation and improved blood glucose, with increases in time in range (TIR) by the continuous glucose monitoring (CGM) method [10,11]. Furthermore, the combination of SGLT2i and incretin-based therapies can be expected to improve both overeating and increased hepatic glucose output.

High-carbohydrate diets, which are common in Asia, can lead to chronic high blood glucose and metabolic abnormalities, especially if too many refined carbohydrates such as white rice are consumed [12]. According to a 2010 survey, the energy ratio of carbohydrate intake to that of all three macronutrients was 59.4% in Japanese people, but in daily practice, there are large differences among individuals [13]. It is worthwhile to assess the effects of carbohydrate ingestion and combinations of drugs to prevent diabetic complications [14,15].

SGLT2i therapy has been shown to produce glucose-dependent urinary glucose excretion and improve blood glucose levels in the presence of high carbohydrates [16,17]. DPP4i have also been reported to improve blood glucose under high-carbohydrate diets [18]. Although the combination of the two drugs has been shown to reduce blood glucose levels, the effects of high- or low-carbohydrate intake in the outpatient setting of the two drugs have been undetermined.

In this study, Hi-Speed Food Analysis was used to assess the exact intake of carbohydrates, and the glucose-lowering effects of luseogliflozin, an SGLT2i, and incretin modulators were observed under high- and low-carbohydrate intakes. The author aims to clarify the effect of combined therapy on blood glucose under high-carbohydrate intake.

## Materials and methods

Subjects

Patients aged ≤75 years with hemoglobin A1C (HbA1C) (National Glycohemoglobin Standardization Program (NGSP)) ≥ 6.8% who regularly visited the Gendai Clinic (Kitakyushu City, Japan) from September to December 2017 were screened. The exclusion criteria were patients with type 1 diabetes, hypersensitivity to SGLT2i, severe infections, and trauma, patients already on SGLT2i and perioperative period, pregnant and lactating patients, and patients excluded at the physician’s discretion. Before the patients were recruited, they were asked whether they could attend serial two-week visits and whether they agreed to the study protocol.

Patient data were masked and stripped of personal information. Under the established protocol, the participants could withdraw from the study freely. All participating patients agreed to the study protocol.

All participants were given an explanation of the study using the protocol in order to obtain their informed consent. In addition, participants were informed of their ability to opt out by documents posted on an announcement board in the clinic.

A total of 37 people with diabetes younger than 75 years old with HbA1C (NGSP) > 6.8% were enrolled. Two were excluded because they could not make frequent visits. The characteristics of the participants are shown in Table 1. A total of 35 T2DM patients (29 male and six female) were recruited. Their mean age was 57.7 years, and their mean HbA1C level was 8.3% (Table 1). Eighteen (51%) patients were on insulin treatment, 12 (34%) were taking GLP-1 receptor agonists, 15 (43%) were taking DPP4i, and 20 (57%) were taking other oral hypoglycemic agents.

**Table 1:** Patients’ profiles Values are shown as mean±standard deviation. BMI: body mass index; HbA1C: hemoglobin A1C; EX-CHO: excess carbohydrate (average intake ≥ 180 g); Low-CHO: low-carbohydrate intake (average intake < 180 g); NS: not significant

Subjects	N=35 (male: 29; female: 6)	EX-CHO group (N=21)	Low-CHO group (N=14)	Significance between EX-CHO and Low-CHO
Age (years)	57.7±10.8	57.0±10.4	58.9±11.9	NS (non-paired t-test)
BMI (kg/m^2^)	25.9±3.9	25.7±3.4	26.1±4.7	NS (non-paired t-test)
HbA1C (%)	8.3±1.3	8.1±1.3	8.5±1.3	NS (non-paired t-test)

Study design

All patients were treated with insulin and/or antidiabetic drugs. During the first week of the study, the subjects continued to take the same medication. In the second week, SGLT2i (luseogliflozin 2.5 mg) was added to the treatment without altering the diabetic drug regimen from week 1.

For intermittently scanned continuous glucose monitoring (isCGM), blood glucose levels were measured using the FreeStyle Libre (Abbott Diabetes Care, Alameda, CA, USA) for two weeks. This isCGM was referred to as flash glucose monitoring (FGM). The mean glucose value was measured in the first and second weeks. Using this method, blood glucose was evaluated based on the length of time in the high, moderate, and low glucose ranges [19].

In this study, the glucose level was evaluated by both the glucose concentration in mg/dL and TIR, either in minutes, TIR(min), or as a percentage of the length of time, (TIR(%)). First, the time above range (TAR) with glucose above 140 mg/dL was expressed as TAR(%140) or TAR(min140). TIR with moderate glucose (70-140 mg/dL) was expressed as TIR(%70-140) or TIR(min70-140). Last, the time below range (TBR) with low glucose up to 70 mg/dL was expressed as TBR(%70) or TBR(min70).

In the present study, patients were recruited in 2017; the range of isCGM had two variations, 140 and 180, and a TAR of 140 was used in this study. Even today, TAR140 remains the second choice, depending on the study [20]. There are also reports that TAR140 or lower is associated with a lower risk of complications [21]. For these reasons, TAR140 and TIR70-140 were used in the present study.

All subjects were not restricted in intaking food energy during the two-week observation period. In the first week, they continued their own treatment, and in the second week of the study period, they took an SGLT2i (luseogliflozin) until the end of the second week. The isCGM (i.e., FGM) unit was attached for two weeks. Energy intake and diet records were submitted at the end of the study. All clinical data were extracted from electronic medical records using Dynamics® (Dynamics Corp., Tokyo, Japan).

Food analysis

**Figure 1:**
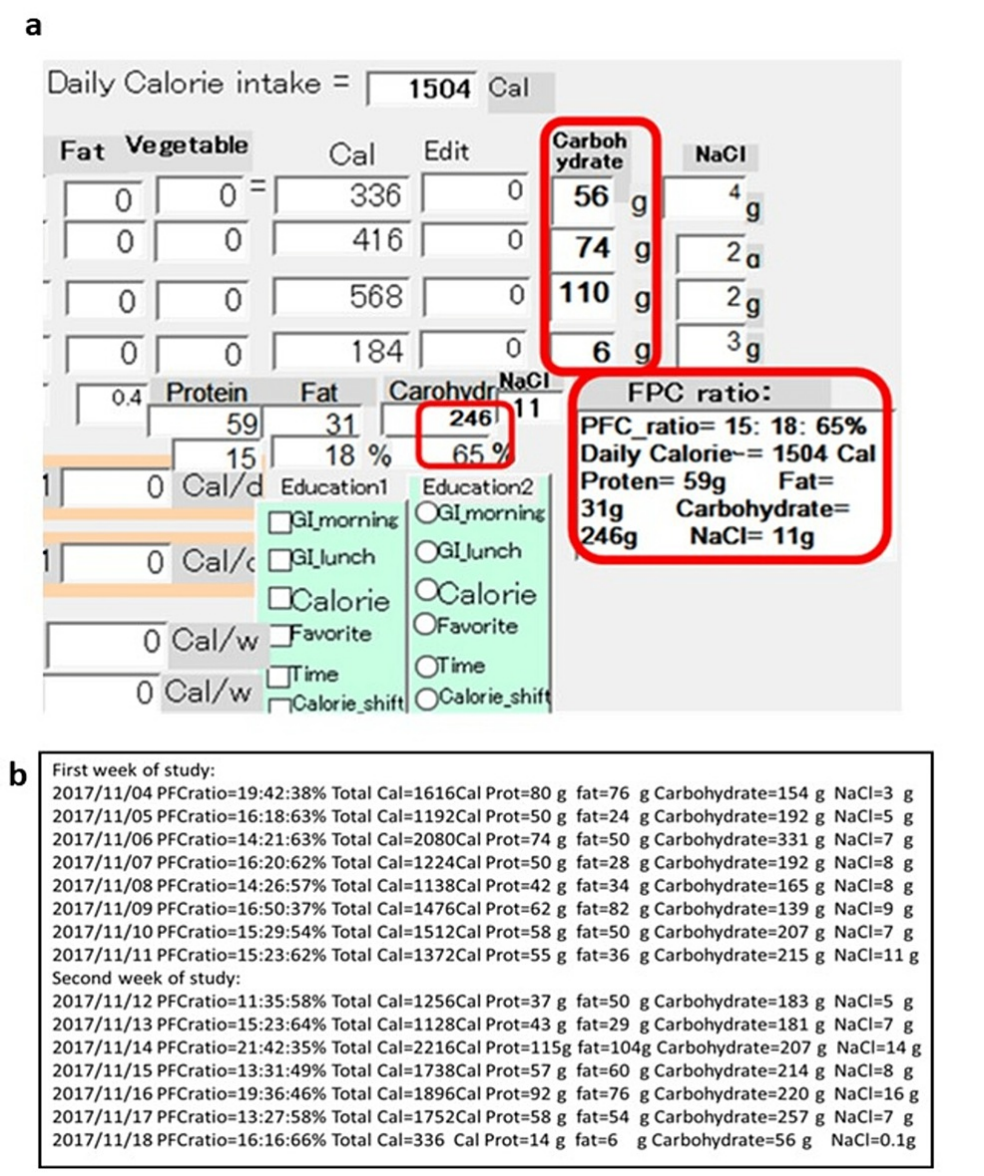
Hi-Speed Food Analysis program and sample results a: Screenshot of the Hi-Speed Food Analysis program. b: Results obtained using Hi-Speed Food Analysis. The daily nutrient composition was recorded in the Dynamics® electronic medical record as a text file. The figure shows each daily protein/fat/carbohydrate (PFC) ratio extracted from the medical record. In this study, weekly average values were used as the amount of personal carbohydrate intake.

Hi-Speed Food Analysis, developed by the author, is a food analysis software application distributed by Support Kitakyushu Corporation (Kitakyushu City, Japan). In brief, this software helps researchers calculate the weight of nutrients, protein, fat, and carbohydrates. The characteristic feature of this software is that it is simple to calculate the three major nutrients and their ratios. This software is preliminarily loaded with various kinds of food analysis results, with the number of preloaded variations approaching nearly 1,000 recipes. Therefore, only a few keyboard commands can lead to a close pattern of nutrient variations. Thus, this may lead to the fewest keyboard touches to obtain precise estimates of nutritional ingredients. Figure 1a is a sample of one candidate with casual daily ingestion who used Hi-Speed Food Analysis. The results showed each nutrient weight and ratio immediately. Figure 1b shows each day’s carbohydrate amount during the isCGM period.

Statistical analysis

Statistical analysis was performed using Statistical Package for the Social Sciences (SPSS) (IBM SPSS Statistics, Armonk, NY, USA). The paired t-test, non-paired t-test, and a general linear model (GLM) were used for analysis. The first- and second-week blood glucose levels were analyzed using Student’s t-test. In this study, the glucose-lowering effects of both an incretin modulator and luseogliflozin were examined with both high- and low-carbohydrate intake. There were many factors in this analysis, such as the ingestion of carbohydrates and the administration of a luseogliflozin and/or incretin modulator. Therefore, the use of a specialized statistical method to clarify the effects of both drugs was needed. GLMs are frequently used and are thoroughly explained in the SPSS textbook. Thus, a GLM is a suitable statistical method and includes not only quantitative independent variables but also qualitative independent variables. Therefore, the four groups of values were analyzed using a GLM, which is appropriate for analyzing multiple factors and taking into account confounding factors. In this study, the factors examined were luseogliflozin, incretin modulators, carbohydrate intake, and TAR(min140). In particular, GLM was used to analyze the effect of carbohydrate intake on the TAR(min140) of luseogliflozin.

Ethical considerations

This study’s protocol was reviewed and approved by the Gendai Clinic Ethics Committee (approval number 191118-01).

## Results

Calculation of carbohydrate intake

In the first week of the study, the mean±standard deviation (SD) carbohydrate intake was 181.0±42.8 g, and in the second week, the carbohydrate intake was 181.0±38.4 g. These values were estimated by Hi-Speed Food Analysis during the two-week observation period. These amounts correspond to 55% of total caloric intake, and there was no difference between the two weeks. Therefore, patients consumed almost the same amount of carbohydrates during the study. These patients were then assigned to two groups based on their carbohydrate intake: one group of patients with excess carbohydrate intake (average intake ≥ 180 g (EX-CHO)), and the other group with low-carbohydrate intake (average intake < 180 g (Low-CHO)). The average value of carbohydrate intake (180 g) was used as the cutoff value. In the EX-CHO group, the mean age was 57 years, and the mean HbA1C was 8.1%; in the Low-CHO group, the mean age was 58.9 years, and the mean HbA1C was 8.5%. There was no significant difference between the two groups (Table 1).

Mean blood glucose, TIR(%70-140), TAR(%140), and TBR(%140) values

Before and after the administration of luseogliflozin, the overall mean±SD blood glucose values measured by isCGM were 156±37 mg/dL and 135±25 mg/dL, respectively. There was a significant difference between the two groups (p<0.001, paired t-test, n=35). The values of TIR(%70-140), TAR(%140), and TBR(%70) improved significantly; TIR(%70-140) improved from 44±22% to 54±21% (p<0.001, paired t-test, n=35), that of TAR(%140) decreased from 52±24% to 38 ± 23% (p<0.001, paired t-test, n=35), and TBR(%70) increased from 3±5% to 6±9% (p<0.05, paired t-test, n=35) (Table 2).

**Table 2:** Mean±SD blood glucose, TIR(%70-140), TAR(%140), and TBR(%140) values before and after the administration of luseogliflozin (n=35) *P-values were calculated using paired t-tests. There were significant differences between the first-week mean plasma glucose and second-week mean plasma glucose, TIR(%70-140), TAR(%140), and TBR(%70) after the administration of luseogliflozin. SD: standard deviation; TIR: time in range; TIR(%70-140): time in range of glucose 70 to 140 mg/dL; TAR(%140): time above range of glucose above 140 mg/dL; TBR(%70): time below range of glucose up to 70 mg/dL

	First week	Second week	P-value*
Mean blood glucose (mg/dL)	156±37	135±25	<0.001
TIR(%140-70)	44±22	54±21	<0.001
TAR(%140)	52±24	38±23	<0.001
TBR(%70)	3±5	6±9	<0.05

EX-CHO group without an incretin modulator

**Figure 2:**
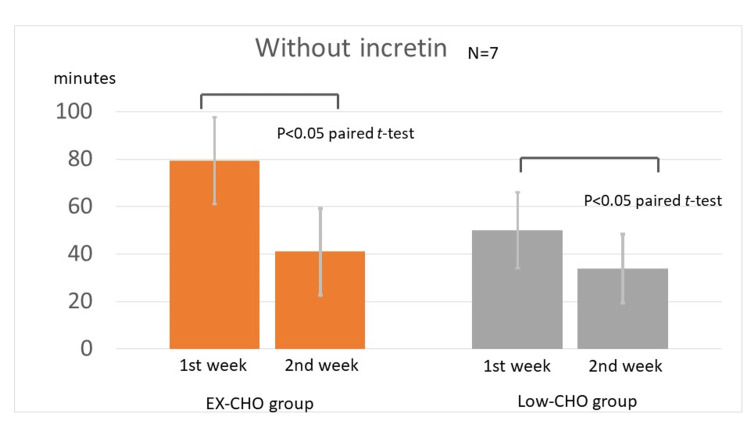
Value of TAR(min140) without incretin before and after the administration of luseogliflozin Without incretin, the effects of luseogliflozin are observed under high-carbohydrate (left panel) and low-carbohydrate (right panel) intakes. The values are expressed as TAR(min140). TAR(min140): time above range (TAR) with glucose above 140 mg/dL; EX-CHO: excess carbohydrate (average intake ≥ 180 g); Low-CHO: low-carbohydrate intake (average intake < 180 g)

The mean±SD TAR(min140) was 79.3±18.3 minutes before the administration of luseogliflozin in the second week and 41.0±16.0 minutes after the administration of luseogliflozin in the second week; the decrease was significant (Figure 2, left panel, p<0.05, paired t-test).

Low-CHO group without an incretin modulator

TAR(min140) was 50.2±18.3 minutes before the administration of luseogliflozin in the second week and 33.8±14.5 minutes after the administration of luseogliflozin in the second week; the decrease was significant (Figure 2, right panel, p<0.05, paired t-test).

EX-CHO group with an incretin modulator

**Figure 3:**
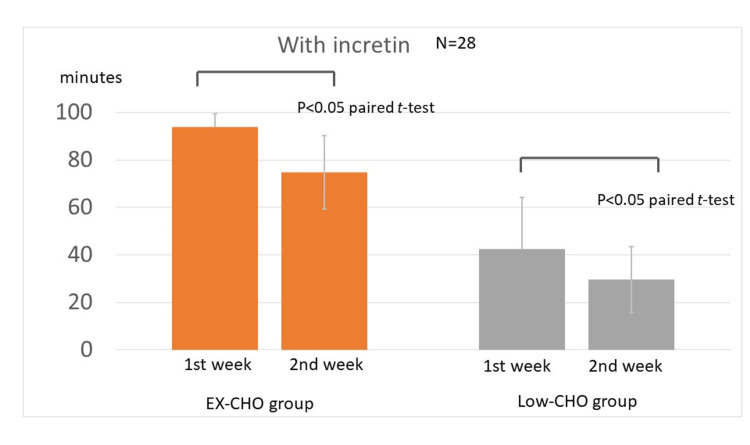
Value of TAR(min140) with incretin before and after the administration of luseogliflozin With incretin, the effects of luseogliflozin are observed under high-carbohydrate (left panel) and low-carbohydrate (right panel) intakes. The values are expressed as TAR(min140). TAR(min140): time above range (TAR) with glucose above 140 mg/dL; EX-CHO: excess carbohydrate (average intake ≥ 180 g); Low-CHO: low-carbohydrate intake (average intake < 180 g)

Both the GLP-1 receptor agonist group and the DPP4i group were included in both the EX-CHO group and the Low-CHO group. TAR(min140) was 94.0±5.6 minutes before the administration of luseogliflozin in the second week and 74.8±21.5 minutes after the administration of luseogliflozin in the second week; this decrease was statistically significant (Figure 3, left panel, p<0.05, paired t-test).

Low-CHO group with an incretin modulator

TAR(min140) was 42.6±15.5 minutes before the administration of luseogliflozin in the second week and 29.7±13.9 minutes after the administration of luseogliflozin in the second week; the decrease was significant (Figure 3, right panel, p<0.05, paired t-test).

Overall subjects without an incretin modulator

**Figure 4:**
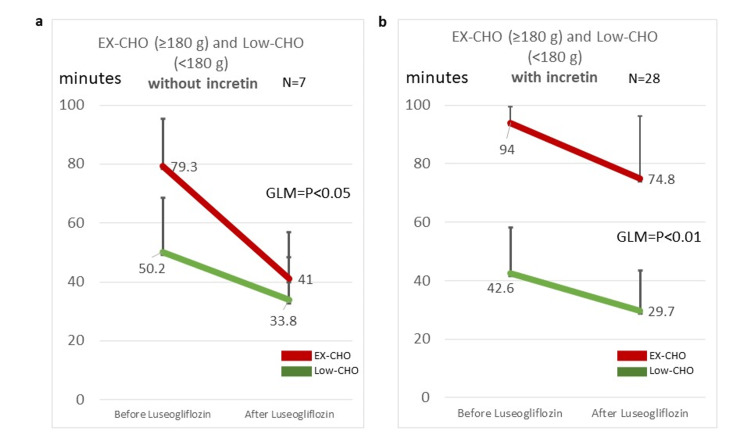
GLM analysis with or without incretin and with or without high-carbohydrate intake or low-carbohydrate intake a: Effects of luseogliflozin on TAR(min140) under the condition of high and/or low carbohydrate without incretin on two-dimensional GLM by SPSS. b: Effects of luseogliflozin on TAR(min140) with incretin on two-dimensional GLM by SPSS. TAR(min140): time above range (TAR) with glucose above 140 mg/dL; EX-CHO: excess carbohydrate (average intake ≥ 180 g); Low-CHO: low-carbohydrate intake (average intake < 180 g); GLM: general linear model; SPSS: Statistical Package for the Social Sciences

There were significant decreases in TAR(min140) by luseogliflozin both with and without an incretin modulator. However, this result did not mean that the actions of luseogliflozin and an incretin modulator were additive. To clarify the effect of luseogliflozin without an incretin modulator, a two-dimensional (2D) GLM was used to test the interaction. The results showed that there was a significant interaction among EX-CHO, Low-CHO, and luseogliflozin administration in the effect on TAR(min140) (Figure 4a, p<0.05, 2D GLM). In other words, carbohydrate intake was thought to be a confounding factor.

Overall subjects with an incretin modulator

Similarly, 2D GLM was used to analyze the interaction with an incretin modulator. The results showed that there was a significant interaction among EX-CHO, Low-CHO, and luseogliflozin administration in the effect on TAR(min140) (Figure 4b, p<0.01, 2D GLM). In other words, carbohydrate intake was thought to be a confounding factor.

## Discussion

In the present study, Hi-Speed Food Analysis was used to accurately assess the intake of carbohydrates, and the glucose-lowering effects of luseogliflozin, an SGLT2i, and incretin modulators were examined under high- and low-carbohydrate intake conditions.

Over the two-week period, the results showed no significant differences between before and after administration of luseogliflozin with regard to carbohydrate intake. Luseogliflozin was administered in the second week, and the mean blood glucose, TIR, and TAR were improved, showing a clear glucose-lowering effect.

Furthermore, the glucose-lowering effects of both an incretin modulator and luseogliflozin were evaluated in the present study with high- and low-carbohydrate intakes. Because there were many factors to consider, such as carbohydrate intake, the administration of luseogliflozin, and/or an incretin modulator, a specialized statistical method was used to clarify the effects of the drugs. An analysis using the GLM was performed to analyze the effect of carbohydrate intake on the TAR(min140) of luseogliflozin. The results showed that there was an interaction between luseogliflozin administration and carbohydrate intake both with and without an incretin modulator. Carbohydrate intake was thought to be a confounding factor.

It is rather difficult to calculate the amount of carbohydrates in an ordinary diet, especially in outpatients, where it takes considerable time and effort to assess the total caloric intake of an individual. However, it is very easy to calculate carbohydrate intake using Hi-Speed Food Analysis, which was developed by the author, as observed in the evaluation of outpatients, where it was used to check the total carbohydrate intake in a diabetes outpatient clinic.

The diabetic subjects who participated in this study were free to consume their routine diet for two weeks with no conditions related to food intake. The total daily carbohydrate intake in the first week was almost the same as that in the second week. To evaluate the effect of a drug, it is very important to maintain the same level of carbohydrate intake.

Matsuba et al. reported that canagliflozin increased caloric intake in patients with T2DM without changing the energy ratio of the three major nutrients [22]. Horie et al. reported that there was increased sugar intake as a form of compensatory hyperphagia in patients with T2DM on dapagliflozin treatment [23]. However, in the present study, during the two-week observation period, the results showed no significant differences in carbohydrate intake before and after the administration of luseogliflozin.

The blood glucose level was checked using the isCGM method, which is useful for calculating the timing of blood glucose changes [24]. Recently, a time range method was adopted to estimate the efficacy of blood glucose-lowering. When interpreting isCGM, the concepts of TIR, TAR, and TBR are useful tools and serve as new metrics related to clinical outcomes [19].

In the present study, luseogliflozin was administered in the second week, and the mean blood glucose, TIR, and TAR were improved, showing a clear glucose-lowering effect (Table 2). TBR(%70) increased significantly from 3±5% to 6±9%. However, the significant increase in TBR(%70) did not indicate hypoglycemic events. There are many reports of isCGM values being lower than levels measured by other methods, especially in the lower range of blood glucose [25]. Therefore, patients having short episodes of TBR do not always indicate hypoglycemia but could show good control.

Luseogliflozin with or without an incretin modulator had a blood glucose-lowering effect in both the EX-CHO and Low-CHO groups. In addition, luseogliflozin had a glucose-lowering effect with and without an incretin modulator (Figure 2 and Figure 3). The incretin modulator reduced high blood glucose concentrations by increasing insulin secretion in diabetic subjects. The mechanism of action of the blood glucose-lowering effect exerted by luseogliflozin involves blocking the action of the SGLT2 transportation system in target cells. Thus, both drugs exerted blood glucose-lowering effects through different actions in diabetic subjects. In addition, incretin has an amplifying effect on insulin secretion in diabetic subjects [26]. However, the effects of carbohydrate intake on the actions of SGLT2i and incretin modulator have not been reported. The results showed that there was an interaction between luseogliflozin administration and carbohydrate intake with and without an incretin modulator. Carbohydrate intake was thought to be a confounding factor (Figure 4). In other words, the glucose-lowering effect of luseogliflozin was more pronounced during the high-carbohydrate intake with or without an incretin administration.

SGLT2i therapy has been shown to produce glucose-dependent urinary glucose excretion and improve blood glucose levels in the presence of high carbohydrates [16,17].

GLP-1 stimulates insulin secretion by activating the protein kinase A (PKA) or Epac2 pathway in a glucose-dependent manner [26], and the hypoglycemic effect of GLP-1 is dependent not only on insulin secretion in response to food intake but also on the inhibition of rapid glucose influx by gastric emptying [6].

Thus, both GLP-1 receptor agonists and luseogliflozin act on different sites in diabetic subjects. This may well explain the enhancement of the effect by high-carbohydrate intake. Both incretin modulators and luseogliflozin decrease blood glucose levels, and each effect was observed by a different method. Neither glucagon nor proinsulin levels were measured in the present study, but Seino et al. reported no significant change after the administration of luseogliflozin [27]. Thus, this might not have been a major issue in the present study. Therefore, the present two agents appeared to act additively in lowing blood glucose levels in patients on a high-carbohydrate diet. Thus, these results indicate that the two drugs have an additive glucose-lowering effect.

Limitations

First, this was a single-facility prospective study in Japan, with a small number of participants who were recruited in a short period. There was a large proportion of males in this study as the ratio of males is larger than that of females in our clinic, and this is consistent with the Japanese diabetes outpatient department.

The number of participants in our study is small to firm conclusions regarding the “additive effect of luseogliflozin on glucose-lowering effect.” The number of participants in the group without incretin is very small compared to the number of those in the group with incretin. Thus, further large-scale studies are warranted. However, we frequently encountered this phenomenon in real outpatient settings.

In Asian countries, it is popular to treat with incretin. So, we observed the paradigm shift in the treatment of diabetes in Japan [28]. It might be possible that luseogliflozin additively affects the glucose-lowering effect of an incretin modulator in a high-carbohydrate diet.

Second, although it is generally difficult to calculate the daily intake of carbohydrates accurately for outpatients, daily carbohydrate intake was simply calculated by Hi-Speed Food Analysis in the present study.

The cutoff values of carbohydrate intake (180 g) derived from the average values of this study were not derived from any criteria. However, Hi-Speed Food Analysis is a program developed by the author and cannot be compared to existing studies.

Third, a potential limitation is the accuracy of isCGM values. The term isCGM replaced FGM (Abbott Diabetes Care), which had been used for some time. The characteristic feature of isCGM is that it can be performed for two weeks. However, by the end of the second week, the values reported by the sensor are lower than those at the start of the first week [29]. Thus, to investigate the effects of drugs, it is recommended that overestimation be avoided. In the present study, TBR showed little increase, but this low value does not always mean hypoglycemia. On the contrary, a slightly lower level of the isCGM value sometimes showed the best range of blood glucose obtained by self-monitoring. Because TBR is expressed as a time or percentage in real-world clinical practice, it is necessary to estimate it more accurately; however, it is a very useful tool to investigate blood glucose level trends over two weeks. Even with these limitations, this study provides information for the further evaluation of the useful effects of luseogliflozin.

Finally, one feature of luseogliflozin is that it should increase urinary glucose excretion. It has been very difficult to persuade participants to check their urine every day. Urinary excretion of glucose was examined qualitatively, but not quantitatively, in the present study. This study focused mainly on the glucose-lowering effects of luseogliflozin treatment with carbohydrate intake.

## Conclusions

The effects of luseogliflozin were observed using isCGM in all patients with type 2 diabetes. In the first group, the observed number was small, but the glucose-lowering effect was observed without the use of an incretin modulator, and this effect was enhanced by a high-carbohydrate diet. In the second group, the glucose-lowering effect was observed with the use of an incretin modulator, and this effect was enhanced by a high-carbohydrate diet. This result suggests that, clinically, these drugs are effective both in monotherapy and in combination therapy.

Furthermore, these glucose-lowering effects were stronger with high-carbohydrate intake than with low-carbohydrate intake, as shown by GLM analysis (SPSS).

In this study, the observed number was small; however, combined treatment with an incretin modulator and luseogliflozin had an additive effect in high- versus low-carbohydrate intake, indicating the possible effectiveness of the combined therapy.
